# Evidence for a Negative Cooperativity between eIF5A and eEF2 on Binding to the Ribosome

**DOI:** 10.1371/journal.pone.0154205

**Published:** 2016-04-26

**Authors:** Danuza Rossi, Natalia M. Barbosa, Fabio C. Galvão, Paulo E. G. Boldrin, John W. B. Hershey, Cleslei F. Zanelli, Christopher S. Fraser, Sandro R. Valentini

**Affiliations:** 1 School of Pharmaceutical Sciences, UNESP - Univ Estadual Paulista, Department of Biological Sciences, Araraquara, SP, Brazil, 14801; 2 Department of Molecular and Cellular Biology, University of California Davis, Davis, CA, United States of America, 95616; The John Curtin School of Medical Research, AUSTRALIA

## Abstract

eIF5A is the only protein known to contain the essential and unique amino acid residue hypusine. eIF5A functions in both translation initiation due to its stimulation of methionyl-puromycin synthesis and translation elongation, being highly required for peptide-bound formation of specific ribosome stalling sequences such as poly-proline. The functional interaction between eIF5A, tRNA, and eEF2 on the surface of the ribosome is further clarified herein. Fluorescence anisotropy assays were performed to determine the affinity of eIF5A to different ribosomal complexes and reveal its interaction exclusively and directly with the 60S ribosomal subunit in a hypusine-dependent manner (K_i_^60S-eIF5A-Hyp^ = 16 nM, K_i_^60S-eIF5A-Lys^ = 385 nM). A 3-fold increase in eIF5A affinity to the 80S is observed upon charged-tRNA_i_^Met^ binding, indicating positive cooperativity between P-site tRNA binding and eIF5A binding to the ribosome. Previously identified conditional mutants of yeast eIF5A, eIF5A^Q22H/L93F^ and eIF5A^K56A^, display a significant decrease in ribosome binding affinity. Binding affinity between ribosome and eIF5A-wild type or mutants eIF5A^K56A^, but not eIF5A^Q22H/L93F^, is impaired in the presence of eEF2 by 4-fold, consistent with negative cooperativity between eEF2 and eIF5A binding to the ribosome. Interestingly, high-copy eEF2 is toxic only to eIF5A^Q22H/L93F^ and causes translation elongation defects in this mutant. These results suggest that binding of eEF2 to the ribosome alters its conformation, resulting in a weakened affinity of eIF5A and impairment of this interplay compromises cell growth due to translation elongation defects.

## Introduction

eIF5A was initially classified as the eukaryotic translation initiation factor 5A due to its stimulation of methionyl-puromycin synthesis [1, 2]. This essential factor is a small acidic protein (17 kDa) composed of two predominantly β-barrel domains. The N-terminal, positively charged, is highly conserved in Archaeas and eukaryotes [3] and is the target of a specific and essential posttranslational modification called hypusination [4–6]. To be modified, deoxyhypusine synthase (DHPS in human and Dys1 in yeast) transfers a 4-aminobutyl moiety from the polyamine spermidine to a specific lysine residue of eIF5A to form deoxyhypusine, in an NAD-dependent manner. Thus, the hydroxylation of the second carbon of the deoxyhypusine residue is catalyzed by deoxyhypusine hydroxylase (DOHH in human and Lia1 in yeast, [7, 8].

Despite the initial evidence for a function in the initiation step of translation, eIF5A was also involved in the translation elongation as loss-of-function mutants of this factor revealed, by polysome profile and ribosome transit time analysis, defects in elongating ribosomes, suggestive of malfunctioning of a canonical translation elongation factor [9, 10]. Translation elongation starts once the ribosomal subunits are joined containing an aminoacyl-tRNA in the P-site. Thus, another aminoacyl-tRNA is positioned into the A site and the peptide bound is formed with the P-site aminoacyl-tRNA by the ribosome itself. Conformational changes impose the translocation of the tRNAs to E and P sites. The essential and well characterized translation elongation factor 2, eEF2, guarantees the complete translocation. This process and factors are very conserved among all organisms but very little is known about the dynamic between each factor [11, 12].

eIF5A has a structural and functional homolog in bacteria, the elongation factor P (EF-P) [13]. Similarly to eIF5A, different species of bacteria undergo post-translation modifications in a corresponding loop of EF-P that is modified in eIF5A: β-lysinylation, an incorporation of lysine in a specific lysine [14–16], and arginine-rhamnosylation, an incorporation of rhamnose in a specific residue of arginine [17].

The crystal structure of EF-P(unmodified)-70S complex was determined [18] and a model of eIF5A binding to the ribosome was proposed based on hydroxyl-radical probing [19]. Both structural data show EF-P/eIF5A binding site between the P and E sites of the 80S with the modified long loop reaching towards the peptidyl-transferase center (PTC). Although no crystal structure is yet available for modified EF-P/eIF5A bound to the ribosome, the positioning revealed by molecular modeling for hypusine, lysyl-lysine and rhamnose-arginine residues in the PTC region is in the vicinity of the CCA-tRNA end, which likely helps to stabilize the P-site peptidyl-tRNA [17]. Moreover, based on the fact that the binding site on the ribosome partially overlaps with that of E-site tRNA, it has been suggested that eIF5A binds to the ribosome only when this site is empty [12], *i*. *e*., in the first round of peptide formation, in agreement with a function in translation initiation [20], and in each cycle of elongation [9, 10].

More recently, it was shown that both eIF5A and EF-P promote peptide-bound formation of proline-rich ribosome stalling sequences [19, 21, 22]. This function has been more extensively studied for EF-P, where ribosome stalling events do not depend only on proline-rich ribosome stalling sequences [21–25], but also on its location relative to the start codon and the translation efficiency due to the ribosome occupancy on the mRNA [25], indicating that other elements contribute to slowing down elongation rates.

In this context, it is not known whether other translation factors influence EF-P/eIF5A relief of stalling ribosomes. Regarding the functional correlation of eIF5A and other translation factors, we have previously shown that eIF5A interacts genetically and functionally with the eukaryotic elongation factor 2 (eEF2), as the growth and protein synthesis defects of the loss-of-function mutant eIF5A^K56A^ are suppressed in the presence of high-copy eEF2 [26].

In order to better understand the dynamics behind the elongation step of translation, we herein demonstrate the effect of eEF2 on eIF5A ribosome binding affinity. Our results show that the binding of eEF2 weakens eIF5A affinity for the 80S, possibly induced by conformation changes. Moreover, *in vivo* data suggest that this interplay between eIF5A and eEF2 binding to the ribosome is important for a balance in translation elongation.

## Materials and Methods

### Sample preparation

The human eIF5A isoform 1 hypusine-containing (heIF5A-Hyp) and unmodified lysine-containing (heIF5A-Lys) were expressed and purified based on previous protocol [27], slightly modified. Briefly, 2 L of BL21(DE3)groES (Takara Bio) cells containing the polycistronic vector co-expressing eIF5A and its modifying enzymes (DHPS and DOHH) were harvested after 16 h of induction at 18°C. Cells were treated as previously described and the first ion exchange chromatography to purify eIF5A was performed using the 5 mL HiTrap-Q column (GE Healthcare). To separate both forms of eIF5A, it was used the 5 mL HiTrap-SP column (GE Healthcare). eIF5A-Hyp and eIF5A-Lys elute approximately at 130 mM of KCl and 230 mM KCl, respectively.

Expression and purification of yeast eIF5A proteins (yeIF5A-wt (wild type), or mutants yeIF5A^C39A^, yeIF5A^C39A,K56A^ and yeIF5A^C39A,Q22H,L93F^) were similar to human proteins, using the polycistronic vector co-expressing the corresponding yeast genes of the modifying enzymes (*DYS1* and *LIA1*). In this case, *TIF51A* was cloned in a vector that allows the addition of a 6xHis-tag sequence in the N-terminus of the protein. Cells were induced, harvested and lysed as previously described in the ice-cold Buffer A’ (50 mM Tris-Cl pH 7.0; 0.1 mM EDTA; 300 mM KCl; 1 mM DTT; 20 mM Imidazole). The lysate was mixed with 5 mL of equilibrated NiNTA resin (Qiagen) and proteins were eluted in Buffer B’ (Buffer A’ containing 250 mM imidazole). Eluate was dialyzed against Buffer C’ (50 mM Tris-Cl pH 7.0; 0.1 mM EDTA; 1 mM DTT) and cationic chromatography in SP column was performed as described.

Yeast ribosomes were prepared based on previous protocols [28, 29] with some modifications. About 30–40 g of cells from early saturated culture of 1–2 OD were immediately frozen in liquid N_2_ and lysed in 40 mL of Lysis Buffer (20 mM Hepes pH 7.5; 100 mM KCl, 5 mM Mg(OAc)_2_, 1 mM DTT, Protease Inhibitors). Cells were splitted into 3–4 round-bottom tubes containing 20 g of glass beads and vortexed during 6 cycles of 1 min ON—1 min OFF, at 4°C. Twenty-five mL of clarified lysate were loaded on the top of a 20% sucrose cushion (20 mM Hepes pH 7,5; 100 mM KCl; 5 mM Mg(OAc)_2_; 1 mM DTT) in a Beckman Ti45 tube. The purification of ribosomes and the separation of 40S and 60S subunits were performed as previously described [28].

Human ribosomes were purified from HeLa cell extracts exactly as previously described [28]. Human initiator tRNA was *in vitro* transcribed with T7 RNA polymerase and methionylated by *Escherichia coli* methionyl-tRNA synthetase as previously described [28]. Recombinant 6xHis-eEF2 was purified as published [30]. The molarity of tRNAs, proteins and ribosomes were determined by 280 or 260 nM absorbance and their molecular mass.

### Protein labeling with fluorescein

The purified single-cysteine mutant of human eIF5A, eIF5A^C22A,C38A,C73A,C129A,T142C^ (eIF5A^T142C^), was labeled with fluorescein-5-maleimide (fl) (Thermo Scientific) following the published protocol [31]. After the labeling reaction, eIF5A-fl was purified from the free dye by spinning in a 2 mL size exclusion column of 6 kDa (BioRad). The amounts of dye and protein were evaluated by 495 nM absorbance and quantitative dotblot using the wild-type protein as standard. Only the preparation with more than 95% of labeling was used for fluorescence anisotropy assays.

### Separation of ribosomal complexes

The mixture of ribosomes and the proteins of interest were isolated by ultracentrifugation in sucrose cushion following published protocols [32, 33]. Yeast 80S ribosomes were incubated at 0.5 μM or 1.0 μM with stoichiometric amounts of eIF5A-Hyp, eIF5A-Lys or eEF2 in a 25 μL final reaction (20 mM Hepes pH 7.5; 100 mM KOAc; 2.5 mM Mg(OAc)_2_; 1 mM DTT; 15 μM BSA). The reaction was incubated at 30°C for 30 min and rapidly chilled on ice. Only 10% of this volume was reserved as input (I) and 20 μL was layered on the top of a 10% sucrose cushion (100 μL) followed by centrifugation at 200.000 rcf for 1 hr at 4°C (Beckman rotor TLS-55). Then, an aliquot of 20μL from the top of the supernatant (S) was saved and the pellet (P) was resuspended in 20 μL of Milli-Q water. Fractions of I, S and P were analyzed by western blot using polyclonal anti-eIF5A, anti-Rpl5 and anti-eEF2 and fluorescent secondary antibody (Thermo).

### Fluorescence Anisotropy

Binding experiments with fluorescein-labeled eIF5A (eIF5A-fl) were conducted using a VICTOR X5 Multilabel Plate Reader (Perkin Elmer), as previously described [28, 31]. A concentration of eIF5A-fl was maintained at 5–10 nM in each reaction. Ribosomes were titrated for each complex with final concentrations at: 2 μM 40S; 1 μM 60S and 500 nM 80S. For competition assays, the concentration of ribosome was constant at 50 nM with 5 μM of starting competitors concentration. The addition of higher concentrations of either ribosome or competitor did not change the equilibrium inhibition constant values, indicating that the reaction was prepared in saturating conditions. Anisotropy binding reactions were set up in 22 μL final volume in reaction buffer (20 mM Hepes pH 7.5; 100 mM KOAc; 2.5 mM Mg(OAc)_2_; 1 mM DTT; 15 μM BSA) and increasing concentrations of ribosome were added gradually. Twenty μL of each reaction was transferred to a 384-wells plate, and incubated at 30°C for 30 min to reach equilibrium. Fluorescence Polarized light (FP) and total fluorescence were measured and the anisotropy change (*r*) was converted into the fraction of eIF5A-fl bound to the ribosome and fitted to the solution of a quadratic equation describing an equilibrium reaction [31]. Inhibition constants (***K***_*i*_) of non-labeled eIF5As were determined in a similar way to that described above, using a preformed ribosomal complex-eIF5A-fl. The values presented in the Table 1 were obtained from the average of three independent experiments, and the errors represent the standard deviation (SD) of the data. All data are expressed as mean values ±SD and analyzed by two-tailed Student's unpaired t-test. In all tests differences were considered significant at p<0.05.

**Table 1:** Values of mean and standard deviation of the anisotropy change and equilibrium binding constants *K*_*d*_ and *K*_*i*_.

	*Y / H*^a^	*K*_*d*_^b^ (nM)	*K*_*i*_^c^ (nM)	*r*_free_ or *r*_min_^d^	*r*_bound_^e^	Δ*r*_max_^f^
**eIF5A-fl**	H					
Met-tRNA_i_^Met^	H	nc	nd	0.176 ± 0.001	0.178 ± 0.004	0.002 ± 0.001
40S	H	^g^ >1700	nd	0.179 ± 0.007	0.186 ± 0.006	0.006 ± 0.001
60S	H	128 ± 3	16 ± 3	0.173 ± 0.001	0.228 ± 0.006	0.055 ± 0.005
40S+60S	H	13 ± 1	12 ± 0.5	0.180 ± 0.001	0.314 ± 0.001	0.135 ± 0.001
40S+60S + tRNAiMet-Met	H	7 ± 1	^h^ ≤ 5	0.198 ± 0.002	0.304 ± 0.006	0.106 ± 0.004
80S	Y	10 ± 1	^h^ ≤ 5	0.187 ± 0.005	0.287 ± 0.001	0.100 ± 0.005
80S + eEF2	Y	41 ± 1	nd	0.134 ± 0.001	0.259 ± 0.005	0.125 ± 0.005
**60S-eIF5A-fl**	H					
eIF5A-Lys	H	nd	385 ± 18	0.186 ± 0.003	0.262 ± 0.001	0.076 ± 0.003
**80S-eIF5A-fl**	Y-H					
eIF5A	Y	nd	9 ± 1	0.177 ± 0.001	0.247 ± 0.006	0.069 ± 0.006
eIF5A^Q22H/L93F^	Y	nd	569 ± 12	0.182 ± 0.003	0.248 ± 0.007	0.066 ± 0.009
eIF5A^K56A^	Y	nd	189 ± 8	0.168 ± 0.005	0.244 ± 0.006	0.076 ± 0.001
**80S-eIF5A-fl + eEF2**	Y-H					
eIF5A	Y	nd	51 ± 3	0.174 ± 0.001	0.242 ± 0.008	0.067 ± 0.007
eIF5A^Q22H/L93F^	Y	nd	539 ± 53	0.204 ± 0.006	0.237 ± 0.010	0.033 ± 0.005
eIF5A^K56A^	Y	nd	454 ± 40	0.188 ± 0.009	0.241 ± 0.010	0.052 ± 0.005

^a^ Origin of each protein and complex where Y is yeast and H is human;

^b^ Equilibrium binding constant obtained by titrating ribosomal complexes into fixed amount of eIF5A-fl;

^c^ Equilibrium binding constant obtained by titrating eIF5A as a competitor into fixed amount of ribosome-eIF5A-fl;

^d^ Anisotropy values for the free eIF5A-fl or the minimum anisotropy detected for the competition assays;

^e^ Anisotropy of the eIF5A-fl bound to the ribosomal complex in the absence of competitors;

^f^ Maximum anisotropy change obtained by the difference between *r*_free_ or *r*_min_ and *r*_bound_;

^*g*^ Lower limits of detection were not superior of 1 mM;

^*h*^ Upper limits of detection were not inferior of 5 nM;

nd = not determined measurements;

nc = not calculated binding constant.

### Physical interaction by two-hybrid system

The used assay was previously optimized [34]. The L40 strain (SVL86) was transformed with plasmids encoding Gal4-eIF5A (vector pACT, pSV285) and lexA-eEF2 (vector pBTM116, pSV272) to test for the transcription of the reporter genes *HIS3* and *lacZ* [34, 35]. All genes used in the constructions for the two-hybrid system are from yeast.

### GST-pulldown

Wild-type cells (VZL1074) expressing GST (pSV20) or GST-eIF5A (pSV36) were grown in SC-ura + 2% galactose until OD_600_ = 0.8. Cells were collected in chilled bottles and treated with 1% formaldehyde for 30 min at 4°C. To quench the formaldehyde, 125 mM glycine was added to the treated cells and incubated for 5 min. After washing, cells were disrupted in lysis buffer 1x (Tris—HCl 20 mM pH 7.5; KOAc 50 mM; MgCl_2_ 10 mM; DTT 1mM and protease inhibitor cocktail) by vortex agitation with glass beads. The clarified lysate was subjected to RNase A digestion (1 mg extract: 30 μg RNase A) for 30 min at room temperature. A volume of the lysate corresponding to 10 OD_254 nm_ units was loaded onto an analytical 10–50% sucrose gradient to confirm the digestion of polysomes. The remaining extract was subjected to GST-pulldown according to a published protocol [36]. Western blot analysis of input (I), wash (data not shown) and bound (B) fractions from GST-eIF5A and GST was performed using rabbit polyclonal specific antibodies against eIF5A, Rpl5 and eEF2, and an ECL system of detection.

### Total Protein synthesis

The measurement of total protein synthesis of wild-type eIF5A (VZL838) and the mutants eIF5A^K56A^ (VZL987) and eIF5A^Q22H/L93F^ (VZL821), in the absence (pSV65) or presence of high-copy eEF2 (pSV262), was conducted as described previously [26].

### Polysome profile and fraction analysis

The polysome profile of the same strains described above, in the absence or presence of high-copy eEF2, was performed as previously described [37]. Proteins from 500 μL of each fraction were precipitated by the addition of 175 μL of 100% trichloroacetic acid (TCA) (final concentration of 13%). After 1–16 h at -20°C, proteins were pelleted by centrifugation at 20.000 rcf for 20 min at 4°C and washed with 1 mL of pure acetone for 1 h at -20°C. The protein pellet was resuspended into 40 μL of 1X SDS loading buffer and 10 μL was analyzed by SDS-PAGE and western blotting. To better detect the differences between the amount of polysomes from each sample, the area of polysome/monosome fractions was calculated (NIH Image J), using he distance from the lower valley to the highest peak.

## Results

### Mutually exclusive binding of eIF5A and eEF2 to translating ribosomes

**Fig 1:**
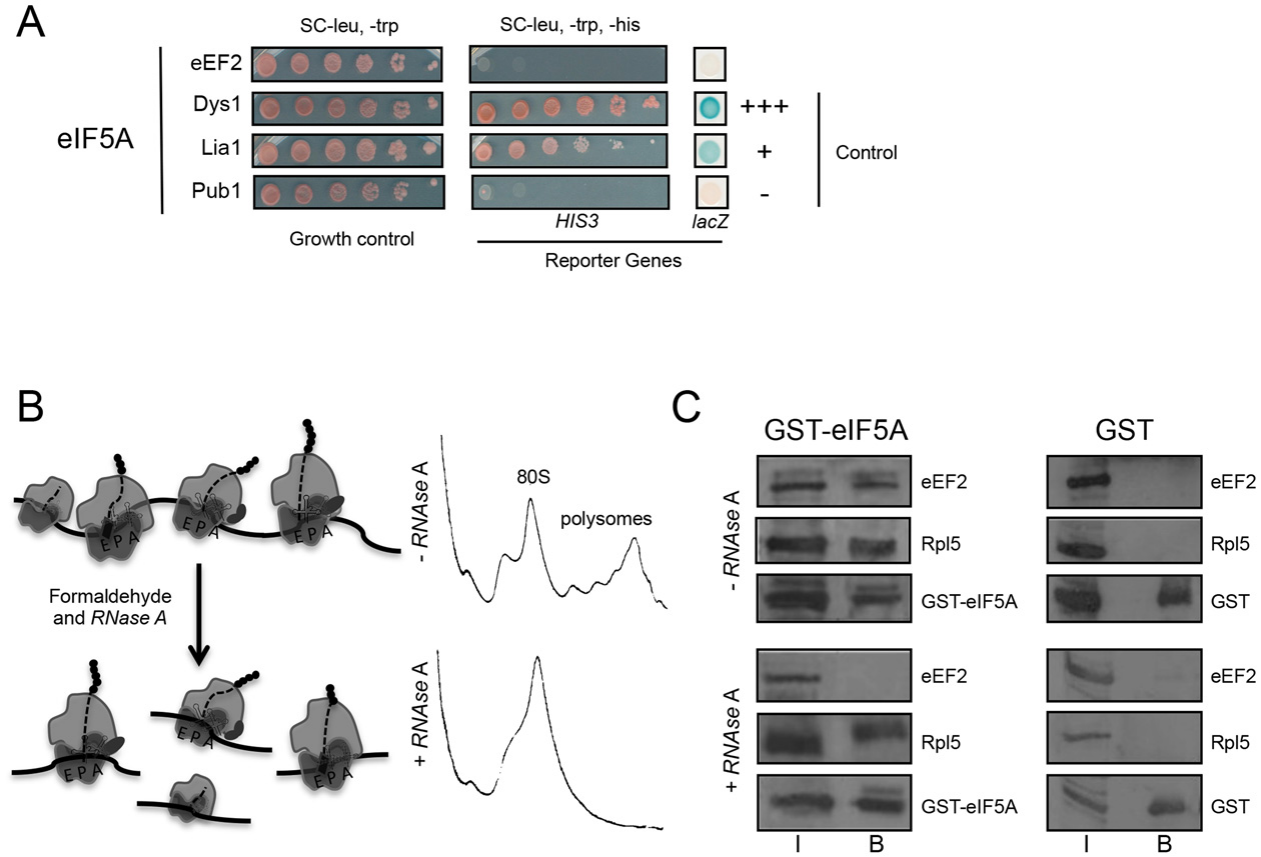
Evaluation of physical interaction between eIF5A and eEF2 by two-hybrid and GST-pulldown of monosomes. (A) Two-hybrid assay. The strain L40 containing pACT-eIF5A was transformed with pBTM116-eEF2, Dys1, Lia1 or Pub1, all yeast proteins. Ten-fold serial dilution of the transformants were grown on SC-leu,-trp (growth control) and SC-leu,-trp,-his (*HIS3* expression). Cells were dropped on nitrocellulose membrane to perform the β -gal activity filter assay (*lacZ* expression). The interaction eIF5A-Pub1 was used as negative control, and eIF5A-Dys1 (strong) or eIF5A-Lia1 (weak) were used as positive controls [8]. (B) Schematic of sample preparation for the *in vivo* GST-eIF5A pull down of monosomes (left panel). Cells were treated with formaldehyde 1% and the extract was treated or not with RN*ase* A to digest polysomes. Polysome profile analysis of the total extract treated or untreated with RN*ase* A (right panel). (C) GST-pulldown assay. Western blot analysis of GST-pull down fractions, input (I) and bound (B), using specific polyclonal antibodies against eEF2, Rpl5 and eIF5A.

We have previously described that eIF5A copurifies with translating ribosomes, which also contain eEF2 [36]. We, then first investigated the possibility of a direct physical interaction between these two yeast factors *in vivo* by two-hybrid analysis in yeast (Fig 1A). The modified yeast strain L40 carries the reporter genes *HIS3* and *lacZ* whose expression is triggered in the system used by the proximity of Gal4-AD (Gal4-activation domain, pACT-eIF5A) and lexA (pBTM116-eEF2). Initially, the levels of fused proteins were tested by western blot to ensure their expression (data not shown). The absence of growth in the selective medium (SC-leu, -trp, -his) and the lack of β-gal activity resultant in the combination of eIF5A and eEF2 (Fig 1A) suggest that these factors do not interact directly or close enough to activate the transcription of *HIS3* and *lacZ*, respectively.

In order to confirm the data suggested by the two-hybrid assay and further evaluate whether this interaction can occur on the same ribosome, we investigated whether the binding of these proteins is dependent on monosomes or polysomes. To this end, a functional GST-eIF5A fusion protein was used in an *in vivo* pull-down assay. Cells expressing GST-eIF5A or GST were first treated with formaldehyde to preserve the translation machinery [38]. Total cell extracts were then incubated in the absence or presence of RNase A to digest the mRNA, converting active polysomes into monosomes (Fig 1B, left panel). The efficiency of mRNA digestion was confirmed, as a single peak of well-preserved 80S monosomes can be seen in the sample treated with RN*ase* A (Fig 1B, right panel). GST-eIF5A pull-downs from untreated, or RNase A treated, lysates are shown in Fig 1C as input (I) and bound (B) fractions. A negative control of GST alone was carried out in parallel to confirm the specificity of the GST-eIF5A pull-down assay. The ribosomal protein L5 (Rpl5) was used to confirm the co-purification of ribosomes by GST-eIF5A. The presence of eEF2 in the fraction B of untreated sample confirms our published data that eIF5A copurifies with ribosome complexes containing eEF2 [36]. Strikingly, eEF2 is no longer detected in the fraction B after the digestion of polysomes with RNase A. This implies that the interaction between eIF5A and eEF2 only occurs when multiple ribosomes on the same mRNA (polysomes) are purified rather than monosomes. This is consistent with negative cooperativity between eIF5A and eEF2 binding to the 80S ribosome.

### eEF2 and hypusine-containing eIF5A are able to displace endogenous eIF5A from the ribosome

**Fig 2:**
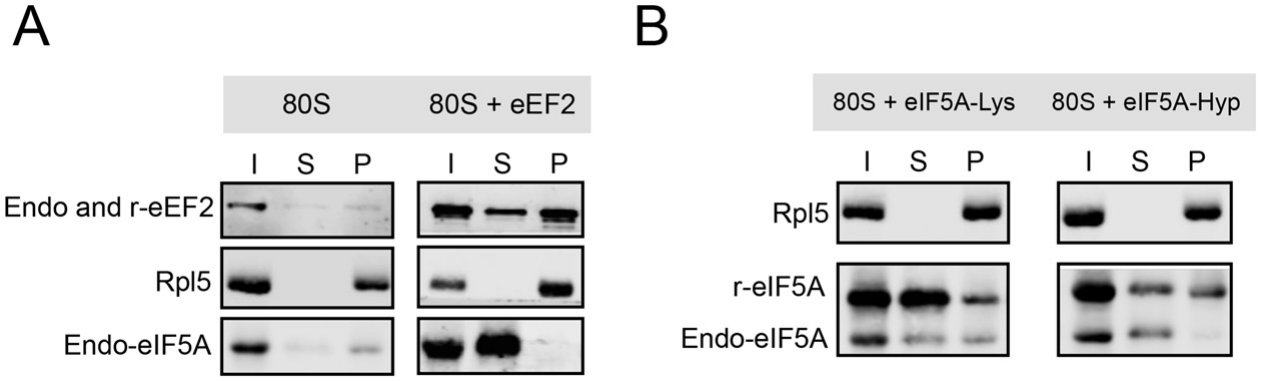
Displacement of endogenous eIF5A from the 80S ribosome by eEF2 or the hypusine-containing eIF5A. (A) Ultracentrifugation of pre-assembled 80S on a 10% sucrose cushion after incubation with recombinant eEF2, eIF5A-Lys or eIF5A-Hyp. (B) Input (I), supernatant (S) and pellet (P) samples were subjected to western blot analysis and the proteins eEF2, eIF5A and Rpl5 were detected using specific antibodies.

Since eIF5A and eEF2 act in the same step of translation but are not detected on the same ribosome, we tested if purified eIF5A and eEF2 can dissociate endogenous eIF5A from purified ribosomes. Pre-assembled 80S ribosomes were purified from yeast lysate and incubated with recombinant eEF2, eIF5A-Lys or eIF5A-Hyp (1 μM of each component) followed by ultracentrifugation in a 10% sucrose cushion. Western blot analysis of the input (I), supernatant (S) and pellet (P) fractions are shown in Fig 2A and 2B. Endogenous eIF5A, eEF2 and Rpl5 were detected in all samples of pre-assembled 80S (I fractions). The P fraction of the sample containing only 80S reveals the endogenous level of eIF5A and eEF2 (Fig 2A, left panel). The addition of recombinant eEF2 to the 80S displaces endogenous eIF5A from the ribosome (Fig 2A, right panel).

To determine if hypusinated eIF5A is required for ribosome binding, the ability of recombinant eIF5A-Lys or eIF5A-Hyp to displace endogenous eIF5A was tested. In this case, the P fractions revealed that both recombinant proteins bind to ribosomes, but only the active form of eIF5A can displace the endogenous eIF5A (Fig 2B). This suggests that the binding affinity of eIF5A-Hyp to the ribosome is higher than eIF5A-Lys, and that the displacement of endogenous eIF5A is due to direct competition. The fact that endogenous eIF5A can also be displaced by the addition of eEF2 confirms that both factors cannot co-exist in the same ribosome at the same time under these experimental conditions.

### The hypusine-containing eIF5A efficiently binds to different ribosomal complexes and its binding affinity is dramatically reduced in the mutants eIF5A^Q22H/L93F^ and eIF5A^K56A^

A fluorescence anisotropy or fluorescence polarization assay was developed to quantitatively evaluate the effect of eEF2 on the affinity of eIF5A binding to the 80S ribosome. This approach is based on the comparison of the rotation (rotation correlation time) of a protein conjugated to a fluorophore when its free (more rotation, less fluorescence polarization) versus when it is bound to large complexes (less rotation, more fluorescence polarization). To that end, a sole residue of cysteine is covalently bound to fluorescein-maleimide. The presence of more than one fluorophore in the same molecule of a protein is prone to reduce fluorescence polarization and generate background issues, leading to the need of data deconvolution and more complicated analysis [28, 31, 39, 40]. In order to generate a robust anisotropy signal, we explored the ribosome-binding of several yeast and human eIF5A-single cysteine mutants, since eIF5A contains several cysteine residues. The use of both yeast and human eIF5A proteins for this purpose is possible because wild-type human eIF5A is able to replace the endogenous yeast eIF5A *in vivo* [6]. Among nine different single-cysteine mutants tested, the human eIF5A^C22A, C38A, C73A, C129S, T142C^ (eIF5A^T142C^) showed the most robust fluorescence anisotropy signal (data not shown) and was selected for further assays.

**Fig 3:**
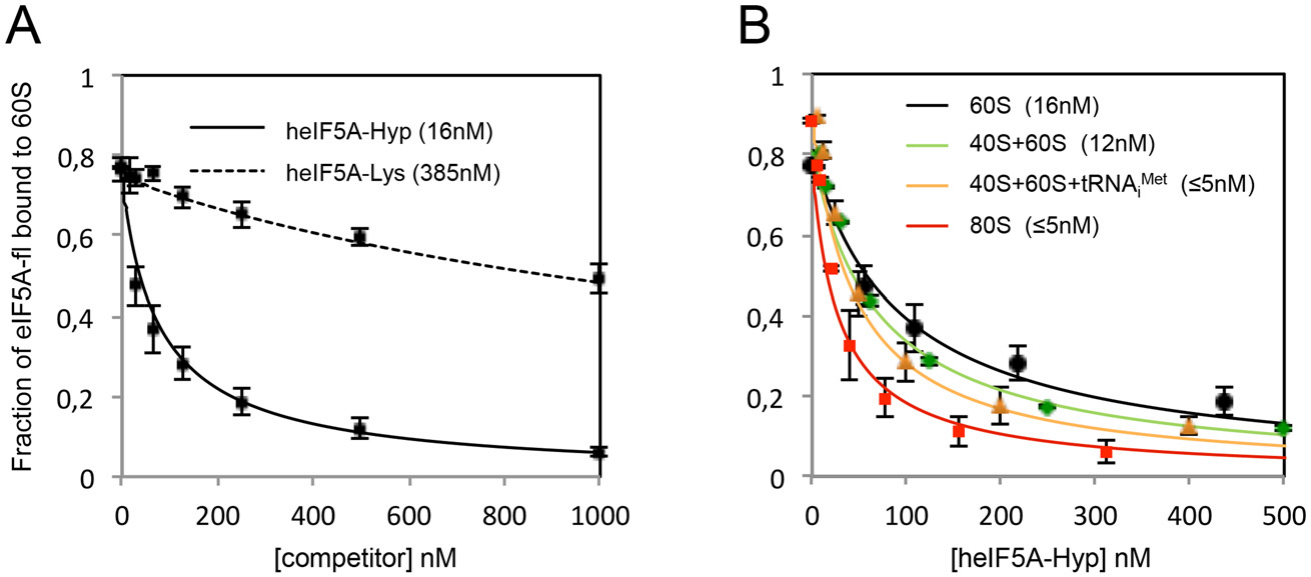
Determination of binding affinity of the hypusine-containing human eIF5A to different ribosomal complexes by fluorescence anisotropy assays. (A) Fluorescence anisotropy changes were measured to calculate the inhibition constant (*K*_*i*_) for the heIF5A-60S interaction. Equilibrium binding of hypusine-containing eIF5A-fl to 60S under the competition with the hypusine and non-hypusine-containing human eIF5A (heIF5A-Hyp and heIF5A-Lys), represented in black curves. (B) Equilibrium binding of hypusine-containing eIF5A-fl to human ribosomal complexes 60S (black), 40S+60S (green), 40S+60S+Met-tRNA_i_^Met^ (orange) and pre-assembled yeast 80S (red) under the competition with heIF5A-Hyp. Each point represents the real measurements from three independent replicates and the error bars correspond to the standard deviation of the data. Inhibition constants, *K*_*i*_ (nM), are shown in parenthesis. Values of anisotropy and equilibrium binding constants (*K*_*d*_ and *K*_*i*_) as well as the standard deviation of the data are noted in Table 1. The error bars represent the standard deviation of the experimental values of anisotropy for the three independent replicates. All data are expressed as mean values ±SD and analyzed by two-tailed Student's unpaired t-test. In all tests differences were considered significant at p<0.05.

To first investigate the binding of human hypusine-containing eIF5A to purified human 60S subunits, the purified subunit was titrated into 5–10 nM of human eIF5A-fluoresceine (eIF5A^T142C^-fl) and the anisotropy changes were measured. Converting *r-*values into the fraction of eIF5A bound to 60S yields an equilibrium dissociation constant (*K*_*d*_) of 128 nM (Table 1). To determine whether the mutations and the presence of the fluorescein affect the interaction with 60S subunits, we also determined equilibrium dissociation constants for an unlabeled eIF5A “inhibitor” (*K*_*i*_) by titrating the non-labeled eIF5A into a fixed amount of pre-formed complex (60S-eIF5A-fl), as described in ‘Experimental Procedures’. In this case, the maximum anisotropy change reveals a high affinity for the non-labeled-eIF5A-60S, *K*_*i*_ = 16 nM (Table 1, solid black line in Fig 3A). The graph in Fig 3A shows the fraction of eIF5A-fl bound to the 60S in the presence of increasing amounts of hypusine (heIF5A1-Hyp) and non-hypusine-containing human eIF5A (heIF5A1-Lys), with the average values for each inhibition constant (*K*_*i*_, nM) shown in parenthesis. The discrepancy between *K*_*d*_ and *K*_*i*_ (8-fold change) suggests that the fluorophore affects the binding affinity of eIF5A to the 60S. Due to this observation, all the following equilibrium binding data were generated using the non-labeled form of human eIF5A^T142C^ in competition assays. Furthermore, the importance of the hypusine residue was also determined by eIF5A-ribosome anisotropy change using the non-hypusine-containing eIF5A^T142C^, as a competitor. Equilibrium binding of non-hypusinated eIF5A^T142C^ to purified 60S subunits was 24-fold weaker than hypusine-containing eIF5A (*K*_*i*_ = 385 nM, dashed black line in Fig 3A), in agreement with the requirement of hypusine for eIF5A function [6, 41].

It is well described for the translation initiation step that the eukaryotic initiation factors (eIFs) binding affinity to the 40S is extremely variable depending on the composition of the ribosomal complexes [42]. Therefore, we investigated binding affinity of eIF5A to 40S, 40S+60S, 40S+60S+Met-tRNA_i_^Met^ or *in vivo* pre-assembled 80S (heterogeneous mixture of yeast 80S). We first determined the equilibrium dissociation constant of human eIF5A-fl to the human 40S+60S, 40S+60S+Met-tRNA_i_^Met^ or yeast pre-assembled 80S complexes (Table 1). Inhibition constants were also determined by titrating non-labeled hypusine-containing human eIF5A (eIF5A-Hyp) into fixed amount of ribosome-eIF5A-fl complexes (Fig 3B and Table 1). The apparent *K*_*i*_ values are not significantly different when the 80S is reconstituted (*K*_*i*_^40S+60S^ = 12 nM), compared to the 60S alone (16 nM). Curiously, the addition of tRNA to the preparation of reconstituted 80S increases the affinity of eIF5A to the ribosome roughly 3-fold (*K*_*i*_^40+60+tRNA^ ≤ 5 nM), which is similar to the affinity of eIF5A to the yeast pre-assembled 80S complexes (*K*_*i*_^80S^ ≤ 5 nM). Due to the fact that these constants are similar to the concentration of the labeled protein (5 nM), the apparent *K*_*i*_ values are likely upper limits.

Molecular modeling based on the structure of the EF-P-70S crystal [18] and 25S rRNA cleavage assays [19] strongly suggest that eIF5A does not interact with the small ribosomal subunit 40S. Titrating 40S instead of 60S under the fixed amount of eIF5A-fl generated very low measurements of anisotropy change (*r*_bound_ and Δ*r*_max_, Table 1), consistent with a lack of interaction between eIF5A and purified 40S subunits. The calculated *K*_*d*_ > 1700 nM is not precise due to the lower limits of detection. Similarly, interaction between eIF5A-fl and aminoacylated-tRNA_i_^Met^ was tested and also revealed very low measurements for anisotropy change (Table 1), also consistent with no interaction between eIF5A and aminoacylated-tRNA_i_^Met^.

Yeast eIF5A was also used in our fluorescence anisotropy assay to determine its binding affinity to purified yeast 80S complexes. The same single-cys mutant (eIF5A^T142C^) was used to preserve the same conditions and allow comparisons with assays performed using human proteins and ribosomes. Since the highest affinity ribosomal complex for human eIF5A was either human 80S+tRNA or yeast pre-assembled 80S (≤5 nM), the last was chosen to evaluate the binding affinity of the following single-cysteine yeast proteins: eIF5A^C39A^ (wt), eIF5A^C39A,K56A^ (eIF5A^K56A^) and eIF5A^C39A,Q22H,L93F^ (eIF5A^Q22H/L93F^).

**Fig 4:**
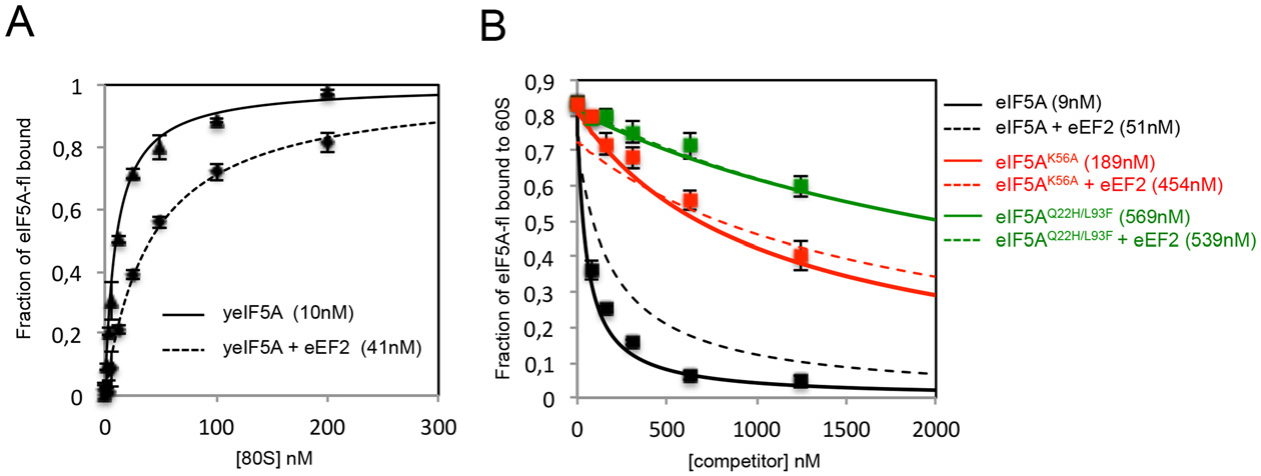
Analysis of the anticooperative effect of eEF2 on eIF5A ribosome binding. (A) Equilibrium binding of eIF5A-fl to the yeast 80S in the presence or not of yeast eIF5A (yeIF5A) and yeast eEF2. (B) Equilibrium binding of eIF5A-fl to the 80S under the competition with yeast eIF5A, eIF5A^K56A^ or eIF5A^Q22H/L93F^, in the presence or not of saturating eEF2. The error bars represent the standard deviation of the experimental values of anisotropy for the three independent replicates. All data are expressed as mean values ±SD and analyzed by two-tailed Student's unpaired t-test. In all tests differences were considered significant at p<0.05. The mean of *K*_*i*_ values is shown in parenthesis. The values of equilibrium binding constants and anisotropy changes are shown in Table 1.

To determine the binding affinity to the 80S, these proteins were titrated individually into a fixed amount of pre-formed 80S-eIF5A-fl, and the change in fluorescence anisotropy was measured to calculate the inhibition constant in equilibrium conditions (Table 1). The fraction of eIF5A-fl bound to 80S and the *K*_*i*_ values are shown in Fig 4B (solid lines). The binding assays revealed a very similar affinity of yeast wt eIF5A compared with human eIF5A for 80S complexes (*K*_*i*_ = 9 nM, black solid line). Interestingly, slightly weaker binding affinities were obtained for the mutants eIF5A^K56A^ and eIF5A^Q22H/L93F^, resulting in decreased ribosome binding affinity by 20 and 60-fold, respectively (*K*_*i*_ = 189 nM, red solid line; *K*_*i*_ = 569 nM, green solid line).

### eEF2 impairs ribosome interaction of wild type eIF5A and eIF5A^K56A^ but not eIF5A^Q22H/L93F^

Having determined the eIF5A equilibrium binding constants to different ribosomal complexes, we evaluated the effect of the presence of eEF2 on eIF5A ribosome binding. The *in vivo* pre-assembled purified 80S was used in the assay as it forms the most stable complex with eIF5A. The pre-assembled 80S ribosome was titrated into fixed amount of eIF5A-fl in the absence or presence of saturating eEF2 (1 μM). The equilibrium binding constant obtained in the presence of eEF2 (*K*_*d*_ = 40 nM) was 4-fold weaker than in the absence of eEF2 (*K*_*d*_ = 10 nM), supporting the hypothesis that eEF2 weakens the interaction of eIF5A with the ribosome (Fig 4A and Table 1). To confirm the saturation condition of eEF2, this assay was carried out also in the presence of 2 μM eEF2 and similar results were obtained (data not shown).

The evidence for an anticooperative binding mechanism between eIF5A and eEF2 for the 80S ribosome was further investigated under conditions where the eIF5A-ribosome interaction is partially lost, *e*.*g*., as in the case of the mutants eIF5A^K56A^ and eIF5A^Q22H/L93F^. Again, changes in anisotropy measurements (Table 1) were detected and Fig 4B shows curves of fraction of eIF5A-fl bound to 80S under the competition with wt (black), eIF5A^K56A^ (red) or eIF5A^Q22H/L93F^ (green), in the absence (solid lines) or presence of eEF2 (dash lines). The equilibrium binding reveals a 3-fold decreased affinity to 80S for the mutant eIF5A^K56A^ in the presence of eEF2, similarly to what was observed for the wt eIF5A (4-fold). Surprisingly, the addition of eEF2 did not cause any effect on eIF5A^Q22H/L93F^ affinity to the ribosome, revealed by similar binding constants (*K*_*i*_≈500 nM). These results reveal that Q22 and L93 play an essential role in the negative cooperative binding of eIF5A and eEF2 to the 80S.

### eEF2 overexpression in the mutant eIF5A^Q22H/L93F^ is toxic due to the impairment in the dynamics behind the elongation step of translation

Given the different effects that yeast eEF2 has on eIF5A^Q22H/L93F^ and eIF5A^K56A^ ribosome binding affinity, we wanted to test if this difference correlates with any effect of eEF2 overexpression on the phenotypic defects of both eIF5A mutants, especially those related to translation.

**Fig 5:**
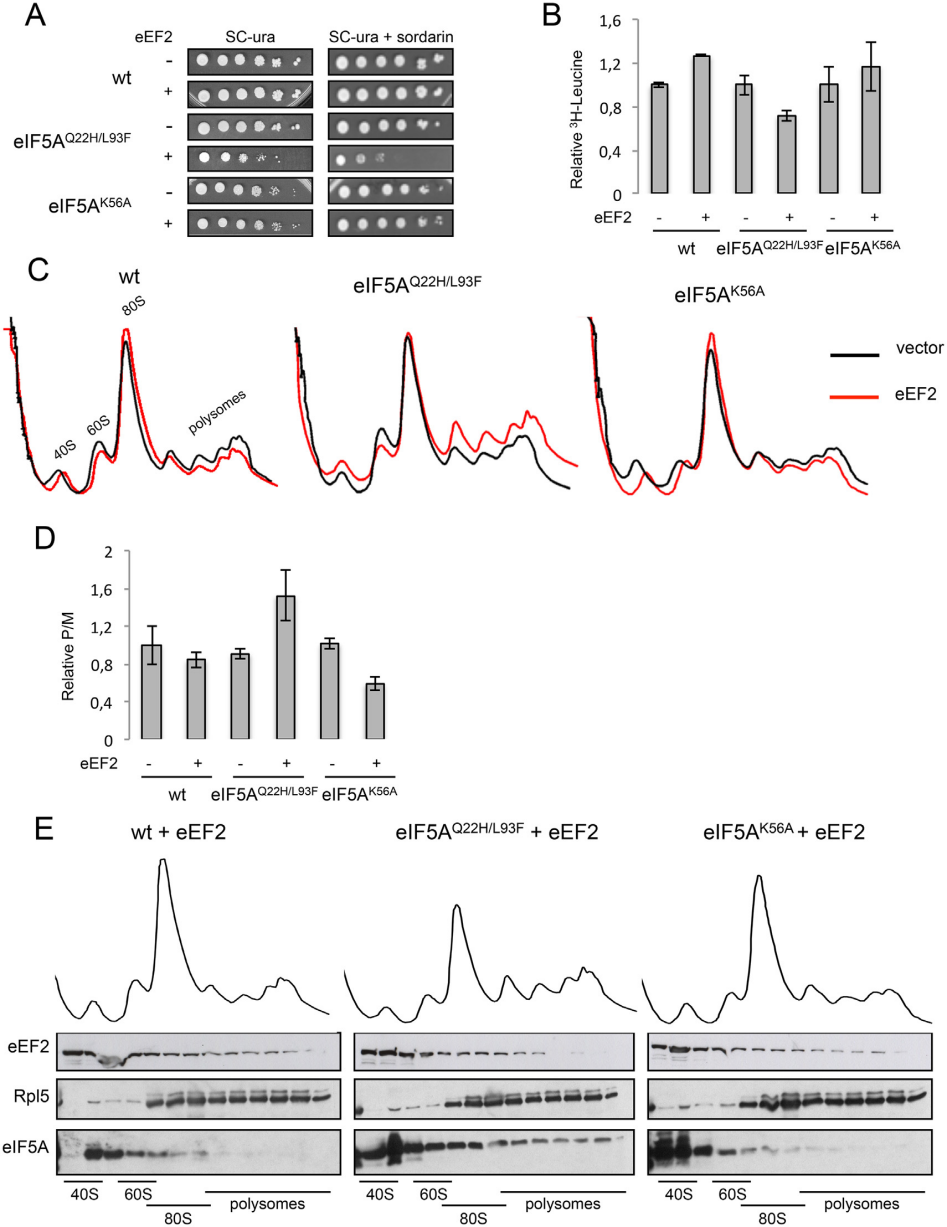
Analysis of the effect of eEF2 overexpression on the phenotypic defects of the mutants eIF5A^K56A^ and eIF5A^Q22H/L93F^ at the permissive temperature (25°C). (A) Ten-fold serial dilutions of wt, eIF5A^Q22H/L93F^ or eIF5A^K56A^, containing empty vector or high-copy eEF2, were grown in SC-ura at 25°C in the presence or not of sordarin (200 ng/mL). (B) Total protein synthesis was measured in the same cells by the incorporation of [^3^H]leucine, relative to the wt strain. (C) and (D) Polysome profile analysis of the same cells was carried out and the quantification of polysome and monosome areas (P/M) were performed to show the levels of translation. (E) Polysome profile fractions of eIF5A^Q22H/L93F^ and eIF5A^K56A^ in the presence of high-copy eEF2 were analyzed by western blot to detect the presence of eIF5A, eEF2 and Rpl5. The error bars in B and D represent the standard deviation of the experimental values for three independent replicates and the data are expressed as mean values ±SD.

Fig 5A shows the overexpression of eEF2 in the mutants eIF5A^Q22H/L93F^ and eIF5A^K56A^ at the permissive temperature (25°C). As already described for eIF5A^K56A^ [26], the effect of eEF2 on growth phenotype at 25°C is almost imperceptible (Fig 5A, left panels). However, overexpression of eEF2 in the mutant eIF5A^Q22H/L93F^ decreases the rates of growth at the permissive temperature (Fig 5A, left panels). Additionally, the sensitivity to sordarin (200 ng/mL), an antifungal that impairs both subunit disassembling and eEF2 activity, is only observed for the mutant eIF5A^Q22H/L93F^ in the condition of eEF2 overexpression (Fig 5A, right panels).

To evaluate whether the defect in cellular growth of eIF5A^Q22H/L93F^ caused by high-copy eEF2 under permissive conditions is due to a defect in translation, we evaluated in wt eIF5A and eIF5A^Q22H/L93F^ or eIF5A^K56A^ mutants the rate of total protein synthesis by incorporation of [^3^H]Leucine. Interestingly, eEF2 overexpression impairs protein synthesis by 30% only in the mutant eIF5A^Q22H/L93F^ (Fig 5B).

Polysome profile analysis of the same cells was carried out to investigate whether the decreased protein synthesis in eIF5A^Q22H/L93F^ mutant is caused by defects in translation elongation. Impairment in translation caused by mutations can be distinguished as translation initiation or translation elongation defects by means of polysome profile analysis comparison between mutant and wild-type. Mutations affecting translation initiation factors reduce the rate of translation initiation, resulting in a simultaneous increase of the 80S peak and decrease in the polysome fraction. On the other hand, mutations affecting translation elongation factors cause the rate of translation initiation to be relatively higher than translation elongation rates, resulting in a concurrent decrease in the 80S and accumulation of polysomes [43, 44]. It has been demonstrated that polysome profiles of eIF5A mutants show increased polysome/monosome ratio (P/M), while eIF4E and eIF3a mutants have their P/M decreased, when compared to the wild-type [9, 10].

The polysome profile of eIF5A-wt, eIF5A^Q22H/L93F^ and eIF5A^K56A^, in the presence or absence of high-copy eEF2, are shown in Fig 5C. The peaks corresponding to the 40S, 60S, 80S and polysomes are indicated in the first profile. The graph in Fig 5D shows the P/M obtained by the quantification of polysome (P) and monosome (M) peak areas, relatively to the wt. It is evident from Fig 5C and 5D that the wt and eIF5A^K56A^ mutant display a pattern completely different from the eIF5A^Q22H/L93F^ mutant in the presence of high-copy eEF2. While there is no significant difference for the wt (although there is a trend for a decrease) or a significant decrease for the P/M of the eIF5A^K56A^ mutant, the eIF5A^Q22H/L93F^ mutant shows a significant increase (p<0.05). This result clearly suggests that eEF2 overexpression leads to impairment of the translation elongation process in the mutant eIF5A^Q22H/L93F^ (Fig 5C and 5D). It is important to note that the results for the eIF5A^K56A^ mutant are in agreement with our previous data [26].

Finally, thirteen fractions from polysome profiles of the mutants eIF5A^Q22H/L93F^ and eIF5A^K56A^ under overexpression of eEF2 were analyzed by western blot and the proteins eEF2, Rpl5 and eIF5A were probed (Fig 5E; S1 Fig). This analysis revealed that eIF5A^K56A^ is present only in the beginning of the profile, similarly to eIF5A-wt (data not shown). The mutant eIF5A^Q22H/L93F^ exhibited again a highly distinct scenario with significantly imprisoned eIF5A in the polysomes even in the presence of eEF2. This result is in agreement with the fact that eEF2 does not exert any effect on the ribosome binding affinity of the mutant eIF5A^Q22H/L93F^ (Fig 4B).

## Discussion

Herein, we addressed the question of how eIF5A and eEF2 functionally interacts to guarantee the functioning of translation elongation. The interaction between eIF5A and eEF2 was initially suggested when they where co-purified *in vivo* by GST-eIF5A pulldown [36]. Later on, a functional relation was described for both factors raising the hypothesis of a positive cooperative effect in the elongation step of translation as high-copy eEF2 suppressed the translation defects of the conditional mutant eIF5A^K56A^ [26]. Guided by that evidence, we investigated the functional interaction between these two translation factors. A two-hybrid assay was used to test for a physical interaction between the yeast factors eIF5A and eEF2, but revealed no direct interaction. The absence of direct physical interaction was expected since the binding sites for eIF5A and eEF2 are structurally distant in the ribosome [45, 19].

Curiously, it was observed that the physical interaction of eIF5A and eEF2 is dependent on polysomes, suggesting that eIF5A and eEF2 do not occupy the same ribosome at the same time [19, 46]. Therefore, we used a non-equilibrium pull-down assay to investigate whether the presence of eEF2 affects the binding of eIF5A to 80S. The addition of eEF2 to pre-assembled 80S complexes containing endogenous eIF5A results in displacement of this factor from the ribosome.

To further investigate whether eEF2 interfere with the binding of eIF5A to the ribosome, we conducted fluorescence anisotropy assays using labeled-eIF5A and different ribosomal complexes to determine the equilibrium binding constants for each complex. It was shown for the first time that the direct interaction of eIF5A to 60S ribosomal subunit occurs without any scaffolding protein and with high affinity (*K*_*i*_ = 16 nM). This interaction is hypusine-dependent, as the non-hypusinated protein displays an equilibrium-binding constant 24-fold higher (*K*_*i*_ = 385 nM). The positively charged hypusine residue promotes an extension of the highly conserved loop that may reach further into the PTC, allowing a close binding to the peptidyl-tRNA 3’-CAA [19]. The biological essentiality of hypusine can be explained by providing much more points of contact and, therefore, stabilizing the protein in a proper position into the ribosome. Consequently, the essential hypusine confers higher activity for eIF5A in translation.

Surprisingly, the eIF5A binding affinity to the reconstituted 80S ribosome (40S+60S) was quite similar (12 nM). By using a more physiologically relevant pre-assembled 80S complex (80S), it was possible to detect an increase by at least 3-fold in the binding affinity of eIF5A for the ribosome. Importantly, both the 40S+60S+tRNA and pre-assembled 80S complexes likely contain an enriched amount of PRE-state ribosomes containing tRNA in the P-site [47], due to the favorite stability of the tRNAs [48]. This observation strongly suggests that charged-tRNA_i_^Met^ in the P-site strengthens the affinity of eIF5A to the ribosome by increasing the points of contact through the interaction eIF5A-Hyp-charged-tRNA_i_^Met^.

The interaction of human eIF5A with yeast pre-assembled 80S was very similar to the human reconstituted 80S (*K*_*i*_≤5 nM), suggesting that the complementation of yeast eIF5A by the human homolog [6] is due to its high affinity to the ribosome. However, the opposite is not true, as the interaction of yeast eIF5A with human ribosomes was also tested by anisotropy change and revealed much lower affinity (>2000 nM; data not shown). Curiously, it was recently described that the configuration and the interactions of tRNA and ribosome in the P/P and E/E states through the cycles of elongation are significantly divergent between bacteria and mammalian ribosomes [47], which leads to specific events during translation elongation in these organisms. Therefore, some of these differences could modify the interaction between eIF5A and tRNA/ribosome in different organisms and explain the fact that yeast eIF5A does not bind very well to human ribosome.

After determining in the human system the binding affinities of eIF5A to different ribosomal complexes, it was then determined the affinity of yeast eIF5A protein in the yeast 80S complex. As expected, the binding affinity of wild-type yeast eIF5A to the yeast 80S was similar to that determined for human (*K*_*i*_ = 9 nM). Again, the high affinity of eIF5A to the 80S, confirmed also in the yeast system, underscores an important evolutionarily conserved direct interaction between eIF5A and the ribosome.

Yeast conditional mutants of eIF5A are well known for their defect in cellular growth and translation efficiency under restrictive conditions [3, 9, 26]. In the case of both eIF5A^Q22H/L93F^ and eIF5A^K56A^ mutants, the protein is not depleted when grown at the restrictive temperature, which is in contrast to most other eIF5A mutants [3]. This finding allows us to investigate the loss-of-function even in the presence of the protein. We described for the first time defects for these mutants even under the permissive temperature. Equilibrium binding under the titration of hypusine-containing eIF5A^Q22H/L93F^ and eIF5A^K56A^ revealed a dramatic decrease in the ribosome binding affinity of more than 20-fold when compared to the wt protein. We directly investigated the influence of eEF2 on eIF5A ribosome binding by measuring the anisotropy change of the eIF5A-80S complex in the presence of saturating eEF2. In agreement with the ability of eEF2 to weaken the eIF5A-ribosome binding affinity in non-equilibrium assays, we determined that eEF2 binding to the ribosome promoted a 4-fold reduction in the affinity of eIF5A to the 80S ribosomal complex.

It will be important in future to understand the biological significance of the negative cooperativity between eIF5A and eEF2. It is likely that the dynamic interplay of these essential factors functions to enhance the rate of translation elongation. Assuming the physiological concentration of eIF5A and eEF2 in eukaryotic cells are much higher than the observed affinities [49], the individual factors are likely in saturating amounts, thereby not affecting the final concentration of the active ribosomal complex *per se*. Instead, the changes in affinity may signify important conformational changes on the ribosome to promote all the different stages during elongation, especially during the translation of polyproline stretches. It is worth noting that while the affinity of eIF5A and eEF2 to the pre-assembled 80S is very similar [50] the abundance of both active factors are very different. Considering that the expression of eIF5A is almost 3 times higher than eEF2 [49], and that all eIF5A is modified (active) in normal cells, it is possible that the occurrence of the eIF5A-80S complex may be higher than eEF2-80S, even eEF2 being clearly required in every cycle of translation elongation. Although this work suggests that the presence of eEF2 weakens the eIF5A-ribosome interaction, further work is still needed to address the kinetics between eIF5A and eEF2 and thus understanding the sequential reactions along the translation elongation process.

The proposed model for the dynamic interplay between eIF5A and eEF2 is reasonable considering wt proteins and complexes. However, to validate this model, it was also tested whether the anticooperative effect of eEF2 on eIF5A ribosome interaction would occur with the loss-of-function mutants of eIF5A. Similarly, the wt eIF5A and the mutant eIF5A^K56A^ showed a 3-4-fold decrease in the affinity to 80S in the presence of eEF2. Surprisingly, the eIF5A^Q22H/L93F^ binding affinity was not affected by the addition of eEF2. There results not only strengthen the model but also strongly suggest that the interplay between these factors requires a certain level of binding affinity to ribosome, which is not observed for the eIF5A^Q22H/L93F^ protein.

In order to further validate the model, it was analyzed the *in vivo* effect of overexpression of eEF2 in both eIF5A mutants. As expected, the overexpression of eEF2 does not affect cellular growth and stimulates total protein synthesis and running-off of the polysomes (P/M rates) in wt and eIF5A^K56A^ mutant. Contrarily, eEF2 overexpression causes slow growth phenotype, sensitivity to sordarin, decrease in protein synthesis and accumulation of polysomes in the eIF5A^Q22H/L93F^ mutant. The diverse responses for this mutant in the presence of high-copy eEF2 correlates very well with the biochemical difference in terms of the negative cooperative effect of eEF2 on eIF5A ribosome interaction. The imprisoning of eEF2 in the ribosome caused by sordarin, that also inhibits its tRNA translocation activity, is even more toxic for the eIF5A^Q22H/L93F^ mutant in the presence of high-copy eEF2 than in the absence of sordarin. Interestingly, it was not seem any sensitivity of eIF5A^Q22H/L93F^ in the presence of high-copy eEF2 against other drugs that impair protein synthesis, *e*.*g*. hygromycin, anisomycin, paromomycin and puromycin (data not shown). It strongly suggests that the impairment of protein synthesis and cell surveillance is not due to a general problem in translation but a disturbance on the dynamic between eIF5A and eEF2 and its negative effect on eIF5A ribosome interaction.

As the last attempt to validate the model, we investigated the migration of these factors using sucrose gradients to identify any defects in eIF5A and eEF2 binding capacity to the polysomes *in vivo*. As expected, the mutant eIF5A^Q22H/L93F^ was the only eIF5A protein detected in the late polysomes, when compared to the wt and eIF5A^K56A^ mutant. The lowest binding affinity of eIF5A^Q22H/L93F^ is not in agreement with imprisoned mutated eIF5A in the polysomes. This apparent conflicting data could be explained by the kinetics of the protein *k*_*off*_/*k*_*on*_ that considers not only the free energy difference (ΔG^0^ = *K*_*d*_) between initial and final state, but also the free energy difference ΔG^1^ (*k*_*off*_) and ΔG^2^ (*k*_*on*_) between initial/final state and transition states. The stay-bound state of eIF5A^Q22H/L93F^ disturbs the effect of eEF2 on the stimulation of eIF5A exit when it binds to the ribosome, resulting in lack of the proper dynamic interplay of both factors. This interplay must be an essential key for the elongation rates of translation, and helps to explain why eEF2 is toxic for the mutant eIF5A^Q22H/L93F^ and suppresses the growth and translation defects of the mutant eIF5A^K56A^ [26]. Kinetic studies are still required to explain the fine-tuning of the anticooperative effect of eEF2 on eIF5A ribosome interaction and help to determine the limiting rates for translation elongation.

## Supporting Information

S1 Fig Independent replicate for Fig 5E. Polysome profile fractions of eIF5A^Q22H/L93F^ and eIF5A^K56A^ in the presence of high-copy eEF2 were analyzed by western blot to detect the presence of eIF5A, eEF2 and Rpl5, as described for Fig 5E.
(TIF)

## References

[1] Kemper WM, Berry KW, Merrick WC. Purification and properties of rabbit reticulocyte protein synthesis initiation factors M2Balpha and M2Bbeta. J Biol Chem. 1976;251(18):5551–7. 965377

[2] Benne R, Hershey JW. The mechanism of action of protein synthesis initiation factors from rabbit reticulocytes. J Biol Chem. 1978;253(9):3078–87. 641056

[3] Dias CA, Cano VS, Rangel SM, Apponi LH, Frigieri MC, Muniz JR, et al Structural modeling and mutational analysis of yeast eukaryotic translation initiation factor 5A reveal new critical residues and reinforce its involvement in protein synthesis. Febs J. 2008;275(8):1874–88. 10.1111/j.1742-4658.2008.06345.x 18341589 PMC5278519

[4] Park MH, Joe YA, Kang KR. Deoxyhypusine synthase activity is essential for cell viability in the yeast Saccharomyces cerevisiae. J Biol Chem. 1998;273(3):1677–83. . 9430712 10.1074/jbc.273.3.1677

[5] Park MH, Wolff EC, Folk JE. Hypusine: its post-translational formation in eukaryotic initiation factor 5A and its potential role in cellular regulation. Biofactors. 1993;4(2):95–104. 8347280

[6] Schnier J, Schwelberger HG, Smit-McBride Z, Kang HA, Hershey JW. Translation initiation factor 5A and its hypusine modification are essential for cell viability in the yeast Saccharomyces cerevisiae. Mol Cell Biol. 1991;11(6):3105–14. 1903841 10.1128/mcb.11.6.3105-3114.1991 PMC360154

[7] Park MH, Liberato DJ, Yergey AL, Folk JE. The biosynthesis of hypusine (N epsilon-(4-amino-2-hydroxybutyl)lysine). Alignment of the butylamine segment and source of the secondary amino nitrogen. J Biol Chem. 1984;259(19):12123–7. . 6434537

[8] Thompson GM, Cano VSP, Valentini SR. Mapping eIF5A binding sites for Dys1 and Lia1: in vivo evidence for regulation of eIF5A hypusination. Febs Letters. 2003;555(3):464–8. 10.1016/s0014-5793(03)01305-x WOS:000187458800008. 14675757

[9] Gregio AP, Cano VP, Avaca JS, Valentini SR, Zanelli CF. eIF5A has a function in the elongation step of translation in yeast. Biochem Biophys Res Commun. 2009;380(4):785–90. 10.1016/j.bbrc.2009.01.148 19338753

[10] Saini P, Eyler DE, Green R, Dever TE. Hypusine-containing protein eIF5A promotes translation elongation. Nature. 2009;459(7243):118–21. 10.1038/nature08034 19424157 PMC3140696

[11] Dever TE, Green R. The elongation, termination, and recycling phases of translation in eukaryotes. Cold Spring Harb Perspect Biol. 2012;4(7). 4/7/a013706 [pii] 10.1101/cshperspect.a013706 . 22751155 PMC3385960

[12] Dever TE, Gutierrez E, Shin BS. The hypusine-containing translation factor eIF5A. Crit Rev Biochem Mol Biol. 2014;49(5):413–25. 10.3109/10409238.2014.939608 25029904 PMC4183722

[13] Rossi D, Kuroshu R, Zanelli CF, Valentini SR. eIF5A and EF-P: two unique translation factors are now traveling the same road. Wiley Interdiscip Rev RNA. 2014;5(2):209–22. 10.1002/wrna.1211 . 24402910

[14] Bailly M, de Crécy-Lagard V. Predicting the pathway involved in post-translational modification of elongation factor P in a subset of bacterial species. Biol Direct. 2010;5:3 10.1186/1745-6150-5-3 20070887 PMC2821294

[15] Navarre WW, Zou SB, Roy H, Xie JL, Savchenko A, Singer A, et al PoxA, yjeK, and elongation factor P coordinately modulate virulence and drug resistance in Salmonella enterica. Mol Cell. 2010;39(2):209–21. 10.1016/j.molcel.2010.06.021 20670890 PMC2913146

[16] Peil L, Starosta AL, Virumäe K, Atkinson GC, Tenson T, Remme J, et al Lys34 of translation elongation factor EF-P is hydroxylated by YfcM. Nat Chem Biol. 2012;8(8):695–7. 10.1038/nchembio.1001 . 22706199

[17] Lassak J, Keilhauer EC, Fürst M, Wuichet K, Gödeke J, Starosta AL, et al Arginine-rhamnosylation as new strategy to activate translation elongation factor P. Nat Chem Biol. 2015;11(4):266–70. 10.1038/nchembio.1751 . 25686373 PMC4451828

[18] Blaha G, Stanley RE, Steitz TA. Formation of the first peptide bond: the structure of EF-P bound to the 70S ribosome. Science. 2009;325(5943):966–70. 10.1126/science.1175800 19696344 PMC3296453

[19] Gutierrez E, Shin BS, Woolstenhulme CJ, Kim JR, Saini P, Buskirk AR, et al eIF5A Promotes Translation of Polyproline Motifs. Mol Cell. 2013 10.1016/j.molcel.2013.04.021 . 23727016 PMC3744875

[20] Henderson A, Hershey JW. Eukaryotic translation initiation factor (eIF) 5A stimulates protein synthesis in Saccharomyces cerevisiae. Proc Natl Acad Sci U S A. 2011;108(16):6415–9. 10.1073/pnas.1008150108 21451136 PMC3081013

[21] Doerfel LK, Wohlgemuth I, Kothe C, Peske F, Urlaub H, Rodnina MV. EF-P is essential for rapid synthesis of proteins containing consecutive proline residues. Science. 339 United States 2013 p. 85–8. 10.1126/science.1229017 23239624

[22] Ude S, Lassak J, Starosta AL, Kraxenberger T, Wilson DN, Jung K. Translation elongation factor EF-P alleviates ribosome stalling at polyproline stretches. Science. 339 United States 2013 p. 82–5. 10.1126/science.1228985 23239623

[23] Hersch SJ, Wang M, Zou SB, Moon KM, Foster LJ, Ibba M, et al Divergent protein motifs direct elongation factor P-mediated translational regulation in Salmonella enterica and Escherichia coli. MBio. 2013;4(2):e00180–13. 10.1128/mBio.00180-13 23611909 PMC3638311

[24] Woolstenhulme CJ, Parajuli S, Healey DW, Valverde DP, Petersen EN, Starosta AL, et al Nascent peptides that block protein synthesis in bacteria. Proc Natl Acad Sci U S A. 2013;110(10):E878–87. 10.1073/pnas.1219536110 23431150 PMC3593848

[25] Woolstenhulme CJ, Guydosh NR, Green R, Buskirk AR. High-precision analysis of translational pausing by ribosome profiling in bacteria lacking EFP. Cell Rep. 2015;11(1):13–21. 10.1016/j.celrep.2015.03.014 . 25843707 PMC4835038

[26] Dias CAO, Borges Gregio AP, Rossi D, Galvao FC, Watanabe TF, Park MH, et al eIF5A interacts functionally with eEF2. Amino Acids. 2012;42(2–3):697–702. 10.1007/s00726-011-0985-0 WOS:000299506000027. 21822730 PMC3245752

[27] Park JH, Dias CA, Lee SB, Valentini SR, Sokabe M, Fraser CS, et al Production of active recombinant eIF5A: reconstitution in E.coli of eukaryotic hypusine modification of eIF5A by its coexpression with modifying enzymes. Protein Eng Des Sel. 2011;24(3):301–9. 10.1093/protein/gzq110 21131325 PMC3038461

[28] Fraser CS, Berry KE, Hershey JW, Doudna JA. eIF3j is located in the decoding center of the human 40S ribosomal subunit. Mol Cell. 2007;26(6):811–9. 17588516 10.1016/j.molcel.2007.05.019

[29] Ben-Shem A, Jenner L, Yusupova G, Yusupov M. Crystal structure of the eukaryotic ribosome. Science. 2010;330(6008):1203–9. 10.1126/science.1194294 . 21109664

[30] Jørgensen R, Carr-Schmid A, Ortiz PA, Kinzy TG, Andersen GR. Purification and crystallization of the yeast elongation factor eEF2. Acta Crystallogr D Biol Crystallogr. 2002;58(Pt 4):712–5. . 11914505 10.1107/s0907444902003001

[31] Sokabe M, Fraser CS. Human eukaryotic initiation factor 2 (eIF2)-GTP-Met-tRNAi ternary complex and eIF3 stabilize the 43 S preinitiation complex. J Biol Chem. 2014;289(46):31827–36. 10.1074/jbc.M114.602870 25246524 PMC4231660

[32] Shin BS, Maag D, Roll-Mecak A, Arefin MS, Burley SK, Lorsch JR, et al Uncoupling of initiation factor eIF5B/IF2 GTPase and translational activities by mutations that lower ribosome affinity. Cell. 2002;111(7):1015–25. . 12507428 10.1016/s0092-8674(02)01171-6

[33] Moreno JM, Kildsgaard J, Siwanowicz I, Mortensen KK, Sperling-Petersen HU. Binding of Escherichia coli initiation factor IF2 to 30S ribosomal subunits: a functional role for the N-terminus of the factor. Biochem Biophys Res Commun. 1998;252(2):465–71. 10.1006/bbrc.1998.9664 . 9826553

[34] Thompson GM, Cano VS, Valentini SR. Mapping eIF5A binding sites for Dys1 and Lia1: in vivo evidence for regulation of eIF5A hypusination. FEBS Lett. 2003;555(3):464–8. 14675757 10.1016/s0014-5793(03)01305-x

[35] Vojtek AB, Hollenberg SM. Ras-Raf interaction: two-hybrid analysis. Methods Enzymol. 1995;255:331–42. . 8524119 10.1016/s0076-6879(95)55036-4

[36] Zanelli CF, Maragno AL, Gregio AP, Komili S, Pandolfi JR, Mestriner CA, et al eIF5A binds to translational machinery components and affects translation in yeast. Biochem Biophys Res Commun. 2006;348(4):1358–66. 16914118 10.1016/j.bbrc.2006.07.195

[37] Galvão FC, Rossi D, Silveira WaS, Valentini SR, Zanelli CF. The Deoxyhypusine Synthase Mutant dys1-1 Reveals the Association of eIF5A and Asc1 with Cell Wall Integrity. PLoS One. 2013;8(4):e60140 10.1371/journal.pone.0060140 . 23573236 PMC3613415

[38] Sutherland BW, Toews J, Kast J. Utility of formaldehyde cross-linking and mass spectrometry in the study of protein-protein interactions. J Mass Spectrom. 2008;43(6):699–715. 10.1002/jms.1415 . 18438963

[39] Toseland CP. Fluorescent labeling and modification of proteins. J Chem Biol. 2013;6(3):85–95. 10.1007/s12154-013-0094-5 24432126 PMC3691395

[40] Puljung MC, Zagotta WN. Fluorescent labeling of specific cysteine residues using CyMPL. Curr Protoc Protein Sci. 2012;Chapter 14:Unit14 10.1002/0471140864.ps1414s70 23151742 PMC3501994

[41] Hershey JW, Smit-McBride Z, Schnier J. The role of mammalian initiation factor eIF-4D and its hypusine modification in translation. Biochim Biophys Acta. 1990;1050(1–3):160–2. 2119810 10.1016/0167-4781(90)90159-y

[42] Acker MG, Kolitz SE, Mitchell SF, Nanda JS, Lorsch JR. Reconstitution of yeast translation initiation. Methods Enzymol. 2007;430:111–45. S0076-6879(07)30006-2 [pii] 10.1016/S0076-6879(07)30006-2 . 17913637

[43] Pestova TV, Kolupaeva VG, Lomakin IB, Pilipenko EV, Shatsky IN, Agol VI, et al Molecular mechanisms of translation initiation in eukaryotes. Proc Natl Acad Sci U S A. 2001;98(13):7029–36. 11416183 10.1073/pnas.111145798 PMC34618

[44] Peltz SW, Donahue JL, Jacobson A. A mutation in the tRNA nucleotidyltransferase gene promotes stabilization of mRNAs in Saccharomyces cerevisiae. Mol Cell Biol. 1992;12(12):5778–84. 1448105 10.1128/mcb.12.12.5778-5784.1992 PMC360517

[45] Kaul G, Pattan G, Rafeequi T. Eukaryotic elongation factor-2 (eEF2): its regulation and peptide chain elongation. Cell Biochem Funct. 2011;29(3):227–34. 10.1002/cbf.1740 . 21394738

[46] Spahn CM, Gomez-Lorenzo MG, Grassucci RA, Jørgensen R, Andersen GR, Beckmann R, et al Domain movements of elongation factor eEF2 and the eukaryotic 80S ribosome facilitate tRNA translocation. EMBO J. 2004;23(5):1008–19. 10.1038/sj.emboj.7600102 14976550 PMC380967

[47] Graifer D, Karpova G. Interaction of tRNA with Eukaryotic Ribosome. Int J Mol Sci. 2015;16(4):7173–94. 10.3390/ijms16047173 . 25830484 PMC4425011

[48] Graifer D, Karpova G. General approach for introduction of various chemical labels in specific RNA locations based on insertion of amino linkers. Molecules. 2013;18(12):14455–69. 10.3390/molecules181214455 . 24287984 PMC6269657

[49] Firczuk H, Kannambath S, Pahle J, Claydon A, Beynon R, Duncan J, et al An in vivo control map for the eukaryotic mRNA translation machinery. Mol Syst Biol. 2013;9:635 10.1038/msb.2012.73 23340841 PMC3564266

[50] Sulima SO, Gülay SP, Anjos M, Patchett S, Meskauskas A, Johnson AW, et al Eukaryotic rpL10 drives ribosomal rotation. Nucleic Acids Res. 2014;42(3):2049–63. 10.1093/nar/gkt1107 24214990 PMC3919601

