# Supplementary figures and images for: Evidence for a Negative Cooperativity between eIF5A and eEF2 on Binding to the Ribosome
Source: PLoS One. 2016 Apr 26;11(4):e0154205. doi: 10.1371/journal.pone.0154205 (PMC4845985; doi:10.1371/journal.pone.0154205)

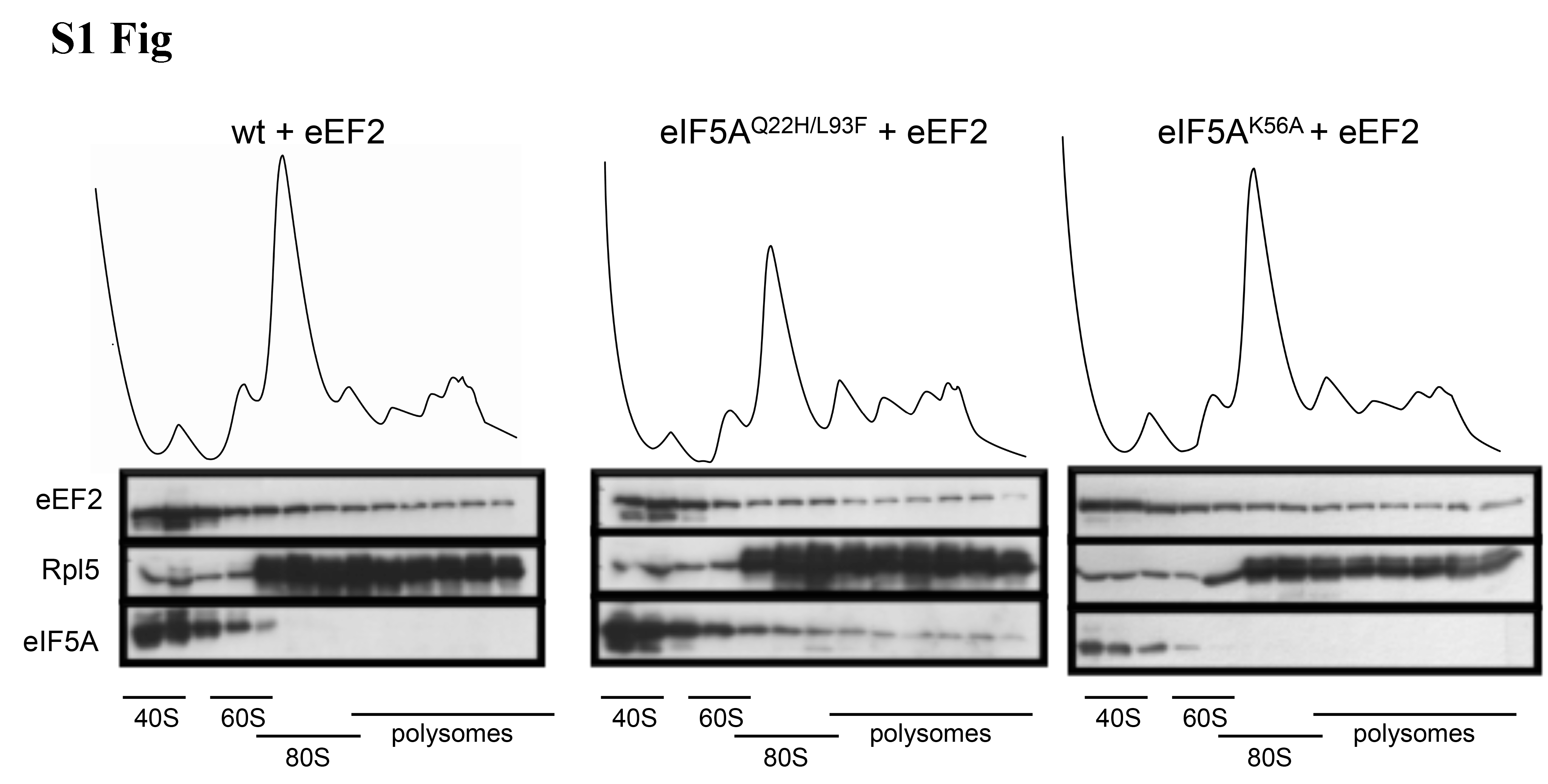

Supplement: S1 Fig — Polysome profile fractions of eIF5AQ22H/L93F and eIF5AK56A in the presence of high-copy eEF2 were analyzed by western blot to detect the presence of eIF5A, eEF2 and Rpl5, as described for Fig 5E. (TIF) [file pone.0154205.s001.tif]
